# Effect of Thermostable Enzymes Produced by Psychrotrophic Bacteria in Raw Milk on the Quality of Ultra-High Temperature Sterilized Milk

**DOI:** 10.3390/foods12203752

**Published:** 2023-10-12

**Authors:** Xue Qin, Jingqi Cheng, Xuehe Qi, Ning Guan, Qing Chen, Xiaoyan Pei, Yujun Jiang, Xinyan Yang, Chaoxin Man

**Affiliations:** 1 Key Laboratory of Dairy Science, Ministry of Education, College of Food Science and Engineering, Northeast Agricultural University, Harbin 150030, China; qingshanmanxue@outlook.com (X.Q.); m13694601123@163.com (J.C.); q1292364109@163.com (X.Q.); chenqingchen163@163.com (Q.C.); yujun_jiang@163.com (Y.J.); 15545116996@163.com (X.Y.); 2 Center for Dairy Safety and Quality, National Center of Technology Innovation for Dairy, No. 1 Jinshan Road, Jinshan Development Zone, Hohhot 010110, China; guanning1@yili.com; 3 Risk Assessment Department, Inner Mongolia Yili Industrial Group Co., Ltd., No. 1 Jinshan Road, Jinshan Development Zone, Hohhot 010110, China; peixiaoyan@yili.com

**Keywords:** psychrotrophic bacteria, UHT milk, thermostable enzymes, quality

## Abstract

Ultra-high temperature sterilized milk (UHT) is a popular dairy product known for its long shelf life and convenience. However, protein gel aging and fat quality defects like creaming and flavor deterioration may arise during storage. These problems are primarily caused by thermostable enzymes produced by psychrotrophic bacteria. In this study, four representative psychrotrophic bacteria strains which can produce thermostable enzymes were selected to contaminate UHT milk artificially. After 11, 11, 13, and 17 weeks of storage, the milk samples, which were contaminated with *Pseudomonas fluorescens*, *Chryseobacterium carnipullorum*, *Lactococcus raffinolactis* and *Acinetobacter guillouiae*, respectively, demonstrated notable whey separation. The investigation included analyzing the protein and fat content in the upper and bottom layers of the milk, as well as examining the particle size, Zeta potential, and pH in four sample groups, indicating that the stability of UHT milk decreases over time. Moreover, the spoiled milk samples exhibited a bitter taste, with the dominant odor being attributed to ketones and acids. The metabolomics analysis revealed that three key metabolic pathways, namely ABC transporters, butanoate metabolism, and alanine, aspartate, and glutamate metabolism, were found to be involved in the production of thermostable enzymes by psychrotrophic bacteria. These enzymes greatly impact the taste and nutrient content of UHT milk. This finding provides a theoretical basis for further investigation into the mechanism of spoilage.

## 1. Introduction

Raw milk possesses a high nutritional content and maintains a moderate pH level, rendering it a favorable habitat for the proliferation of microorganisms. Among these microorganisms, psychrotrophic bacteria are widely recognized as the primary cause of spoilage in milk and dairy products [1]. The International Dairy Federation (IDF) has defined cold-resistant bacteria as those that grow at 7 °C and psychrotrophic bacteria as those that grow below 20 °C [2]. Despite the fact that contamination risks are offset by cold storage [3], psychrotrophic bacteria can still proliferate and become the dominant microorganisms during the storage process [4]. Pasteurization or ultra-high temperature sterilization (UHT) can effectively deactivate psychrotrophic bacteria in milk [5]. However, the production of extracellular protease and lipase by bacteria continues to be a concern for milk quality during processing and storage, resulting in a reduction in its shelf life [6]. The diversity of psychrotrophic bacteria in raw milk has been extensively studied, with the primary species identified in the genera *Pseudomonas*, *Chromobacterium*, *Lactobacillus*, *Clostridium*, *Corynebacterium*, *Streptococcus*, *Flarobacterium*, *Micrococcus*, *Alcaligenes*, and *Enterobacterium* [7]. *Pseudomonas* spp. has received particular attention due to its heat-resistant protease which exhibits exceptional proteolytic ability [8]. *Acinetobacter* spp. has also been recognized as a representative genus associated with fat decomposition [9].

The protease and lipase produced by psychrotrophic bacteria are known for their high heat resistance. Matéos et al. [10] found that even after being treated at 110 °C for 120 s, the residual protease activity can still retain approximately 82.5 ± 5.6%. This is attributed to the strong recovery capability of the thermostable protease. During high-temperature heat treatment, some of the protease denatures and loses its activity. However, as temperatures decrease, the protein structure of the protease slowly returns to normal, leading to the continued deterioration of milk quality [11]. The presence of protease and lipase enzymes can cause quality issues, including rancidity, changes in flavor, sediment formation, and instability in gels, which can result in significant financial losses [12]. Therefore, psychrotrophic bacteria and their thermostable enzymes have become the main focuses of the dairy industry.

Metabolomics is a burgeoning discipline in the post-genomics era that employs quantitative analysis to investigate the intrinsic metabolites found within an organism [13]. Untargeted metabolomics holds significant potential as a valuable tool for identifying the metabolic changes associated with biological activity in specific biological or environmental contexts [14]. This study focuses on investigating thermostable enzymes produced by psychrotrophic bacteria and aims to predict potential quality degradation in milk by analyzing the metabolic signature caused by contamination. The main goal is to extend the shelf life of milk and reduce financial losses caused by psychrotrophic bacteria.

## 2. Materials and Methods

### 2.1. Materials

Reagents including azocasein, diethyl ether, petroleum ether, and 1, 2-propylene glycol were purchased from Solarbio (Beijing, China). Trichloroacetic acid (TCA), p-nitrophenol, methyl red indicator, bromocresol green indicator, methylene blue indicator, Rhodamine B, and 2-methyl-3-heptanone were obtained from Aladdin (Shanghai, China). Commercial whole milk was purchased from a local supermarket. All culture media used in this manuscript were obtained from Hopebio (Qingdao, China). Tributyrin was sourced from Sigma-Aldrich (St. Louis, MO, USA).

### 2.2. Strain Cultivation and Screening of Enzyme-Producing Strains

The 136 psychrotrophic bacteria used in this study were previously isolated from raw milk in Heilongjiang Province [15]. The classification and number of these strains are listed in Table S1. The strains were cultured with a 2% inoculum in lysogeny broth (LB) at 25 °C for 24 h with shaking at 150 rpm/min. This process was replicated twice for further experimentation. To evaluate their ability to produce protease and lipase, all strains were tested on plate count agar supplemented with 6% skimmed milk powder (0.06 g/mL) and tributyrin agar, respectively. The plates were incubated at 7 °C for 10 days. The enzyme ability was indirectly measured by calculating the radius of hydrolysis [16] using the following equation:

R = D − d,(1)

where R (cm) was the radius of hydrolysis; D (cm) was the protease/lipase hydrolysis circle diameter; d (cm) was colony diameter.

### 2.3. Quantitative Assessment of Total Proteolytic and Lipolytic Activity

Strains that exhibited positive protease and lipase production on screening medium were chosen for quantifying their total proteolytic and lipolytic activity. All strains were cultured in LB medium for 24 h on a shaking incubator at 150 r/min and this process was repeated twice. Subsequently, the bacterial suspension was then diluted in 50 mL commercially available UHT milk with a 2% inoculum and incubated at 7 °C for 10 days. Centrifugation was carried out at a speed of 12,000× *g* for 20 min at 4 °C, followed by filtration using a 0.45 μm filter membrane. The resulting supernatant was collected for further use. Proteolytic activity was determined using azocasein (Sigma, USA) as a substrate, following the protocol described by Yuan et al. [17]. A control was established, using 100 μL of sterile PBS instead of supernatant. The proteolytic activity was expressed as the increase in absorption at 366 nm per hour and per milliliter (ΔA/(h·mL)), and each assay was performed in triplicate.

The process of strain culture and enzyme production followed the same procedure as described above. Afterward, centrifugation was conducted at a speed of 15,000× *g* at 4 °C for 20 min, and filtration was carried out using 0.45 μm filter membrane to prevent interference from milk proteins. Lipase activity was measured using p-nitrophenol palmitate (p-NPP) as a substrate, following the method described by Yuan [17]. A control group was included, where 100 μL sterile PBS was used instead of supernatant. The lipolytic activity is expressed as the amount of p-nitrophenol (μmol) released per minute per milliliter (μmol/(min·mL)) of milk sample and each assay was performed in triplicate.

### 2.4. Thermostability of Proteases and Lipases

The methods of strain culture and enzyme production were conducted as previously described. To simulate pasteurization and ultra-high-temperature treatment, 100 μL supernatant samples were subjected to heat treatment in a water bath at 65 °C for 30 min, 90 °C for 16 s and 140 °C for 5 s in an oil bath. Subsequently, the samples were immediately cooled in ice. Proteolytic and lipolytic activity were measured using methods described above. Each assay was performed in triplicate. The heat resistance of protease was calculated by residual activity rate of the enzyme (RA), using the following formula:

(2) $$\mathrm{RA}=\frac{A_{t}}{A_{0}}\times 100,$$

where RA (%) was residual enzyme activity; A_t_ (ΔA/(h·mL)) was the enzyme activity after heat treatment; A_0_ (ΔA/(h·mL)) was the enzyme activity without treatment.

### 2.5. Preparation of Artificially Contaminated Milk Samples

Four presentative strains from four genera (*Pseudomonas*, *Chryseobacterium*, *Lactococcus*, and *Acinetobacter*) were selected among the dominant thermostable enzyme-producing strains for milk sample preparation. The strains were cultured as mentioned above, and the final cell count was ~10^8^ CFU/mL for all of the three strains. Here, 3 mL of an appropriate dilution of the adapted culture was aseptically inoculated in 3 L of whole milk in order to obtain contaminated milk samples at final concentrations of 10^1^, 10^2^, and 10^3^ cfu/mL, respectively. A blank sample consisting of 1 L non-inoculated milk was processed the same way. The milk samples were stored in erlenmeyer flasks at 10 °C for 48 h for strain growth and enzyme production. Then, aliquots (100 mL) of samples were transferred aseptically into 200 mL sterile bottles and immediately batch heated at 97–98 °C for 4 min (with additional 10 min heating time) to destroy microorganism and plasmin. After cooling, the milk sterility was tested and sodium azide was added at 2% in order to inhibit the growth of microorganisms. The samples were sealed and stored in the dark at 25 °C until analysis. Each experiment was conducted in duplicate. During storage, the samples were analyzed every 2 weeks until significant whey separation occurred.

### 2.6. Physicochemical Properties of Milk Samples

#### 2.6.1. Protein and Fat Content

The protein and fat content of the upper and bottom layers of milk samples were determined using the Kjeldahl method and alkaline hydrolysis method, respectively [18,19]. A pipette tip was slowly inserted along the inner wall of the tube until it reached the bottom, and then the same volume was collected from the upper and bottom layers successively.

#### 2.6.2. pH

Changes in pH were measured using a pH meter (Mettler-Toledo, Columbus, OH, USA). A 40 mL aliquot of milk was transferred into glass cuvettes. After calibration, the pH meter electrode was carefully inserted into the milk sample emulsion and the pH value was recorded once it stabilized. Each sample was measured three times.

#### 2.6.3. Taste

Taste analysis was conducted using an electronic tongue (INSENT, Kanagawa Prefecture, Japan). A 35 mL aliquot of liquid milk was injected into the sampling cup of the electronic tongue and sealed at room temperature for 20 min. Before analyzing, the taste sensor of the electronic tongue was soaked in buffer for 24 h. The testing procedure began after the instrument was self-checked and calibrated. Each sample was measured four times, and three stable data were selected for analysis.

#### 2.6.4. Volatile Substances

Volatile substances were determined using the internal standard method. A 10 mL milk sample, 2 g sodium chloride, and 0.58 μL of the internal standard 2-methyl-3-heptanone (0.816 μg/μL) were mixed into the headspace vials. The headspace condition for releasing volatile compounds from the sample involved heating at 40 °C for 30 min. The manual HS-SPME injector was fixed on the SPME carrying device. Samples were extracted by SPME fibers for 30 min and desorbed for 5 min using GC-MS (Shimadzu, Kyoto, Japan). The GC conditions were as follows: the oven was initially set at a temperature of 30 °C and held for 1 min, then programmed to increase to 300 °C at a rate of 5 °C/min. Helium was used as the carrier gas at a flow rate of 1.0 mL/min, and the sample was injected without a shunt.

The mass spectrometer scanned from 50 u to 380 u using the full scan mode. The transmission line and quadrupole temperature were at 250 °C and 150 °C, respectively. The ion source temperature was set at 230 °C and spectra were obtained using electron impact (70 eV). The solvent was delayed for 5 min. Compounds were tentatively identified by comparing their MS spectra with those of members in the library.

### 2.7. Stability of UHT Milk

The stability of UHT milk samples was assessed by analyzing their size, Zeta potential and microstructure. Size measurements were obtained by diluting the samples 50-fold with ultrapure water, while Zeta potential measurements were acquired by diluting the samples 1000 times with a PBS solution (0.01 M, pH 7.3) using a particle size and potential analyzer (Malvern Instruments Ltd., Great Malvern, UK). The particle size distribution analysis was conducted using a polydisperse model with specific conditions: a real refractive index of 1.349, an absorption of 0.001, and a refractive index of the fluid (water) of 1.33 [20]. The refractive index of the Zeta potential was as follows: a dispersed phase of 1.33, a viscosity of 0.8872 cP, and a dielectric constant of 78.5. For observing the fat and protein content, the milk sample was diluted 200 times with ultrapure water and stained with 1% Nile red and 1% Rhodamine B, respectively. The staining procedure involved agitating the sample vigorously and incubating it in a light-restricted environment for 30 min. A 5 µL volume of the mixture was then placed on a cover glass, ensuring there were no bubbles, and examined using an inverted fluorescence microscope (Leica, Wetzlar, Germany) to evaluate the microstructure of the UHT milk.

### 2.8. Mechanism of UHT Milk Quality Change

Prior to metabolomics profiling, the samples were collected and prepared. The samples contaminated by *P. fluorescens*, *C. carnipullorum*, *L. raffinolactis*, and *A. guillouiae* after 7 days were labeled as A1, A2, A3, and A4. The samples without any treatment were labeled as the CK (contrast check) group. The collected milk samples were immediately frozen at −80 °C and stored until analysis. After slowly thawing the samples at 4 °C, an appropriate amount of samples was taken and added to a pre-cooled methanol/ethylene glycol/aqueous solution (2:2:1, *v*/*v*). The mixture was then vortexed and subjected to low-temperature ultrasound for 30 min. After standing at −20 °C for 10 min and centrifugation at 14,000× *g* at 4 °C for 20 min, the supernatant was collected for vacuum drying and further use. The dried samples were redissolved in 100 μL of an aqueous solution of acetonitrile (acetonitrile: water = 1:1, *v*/*v*). The supernatant obtained after vortexing and centrifugation was used for mass spectrometry analysis. The liquid chromatography–mass spectrometry portion of the platform utilized an Agilent 1290 Infinity LC ultra-high performance liquid chromatography system (UHPLC) and an AB Triple TOF 6600 mass spectrometer. The mass spectrometer operated at nominal mass resolution and consisted of an electrospray ionization (ESI) source and a linear ion-trap mass analyzer. The operation and data processing were conducted with the assistance of Shanghai Personal Biotechnology Co., Ltd. (Shanghai, China).

### 2.9. Statistical Analysis

The data were analyzed using a one-way ANOVA with SPSS Statistics 20.0 software (SPSS Inc., Chicago, IL, USA). The results were expressed as mean ± standard deviation. Statistical significance was defined as *p* < 0.05. Multivariate statistical data analysis was established for metabolite data analysis and interpretation. Log transformation and pareto scaling were applied to realize standardization and normalization of generated data independently. Unsupervised statistical analysis was performed by principal component analysis (PCA) in order to classify and discriminate the groups. Based on univariate analysis, all metabolites detected in positive and negative ion modes (including unidentified metabolites) were analyzed. The differential metabolites (FC (Fold Change Analysis) > 1.5 or FC < 0.67, *p* < 0.05) were visualized by volcano maps. The volcano maps and pathway maps were generated from R software (v4.2.1).

## 3. Results and Discussion

### 3.1. Hydrolytic Evaluation of Strains and Heat Resistance of Enzymes

In this study, we examined the capacity of 136 strains of psychrotrophic bacteria to produce protease and lipase. These strains were obtained from 20 raw milk samples, as previously described in our earlier research [15]. The initial evaluation of these strains was conducted by measuring the size of the hydrolysis circles, and the findings are depicted in Figure S1. Our findings revealed that *Chryseobacterium* spp. And *Lactococcus* spp. Exhibited strong proteolytic ability at 7 °C for 10 days, which is consistent with the studies conducted by Vithanage [9], Baur [21], and Sun [22]. On the other hand, *Acinetobacter* spp. demonstrated strong lipolysis ability, while *Pseudomonas* spp. exhibited both proteolytic and lipolytic abilities, which has been confirmed by Xin [23]. Consequently, the top 50% strains with excellent proteolysis or lipolysis were selected for further analysis of their heat resistance. These strains included 34 *Pseudomonas* spp., 14 *Chryseobacterium* spp., 11 *Lactococcus* spp., and 9 *Acinetobacter* spp.

Commercial pasteurization (65 °C for 30 min, 90 °C for 16 s) and ultra-high-temperature instantaneous sterilization (140 °C for 5 s) were simulated to study the heat treatment of the enzymes produced by strains. The remaining activity (RA) of the enzyme was measured to assess the proteolytic and lipolysis characteristics of the enzymes. The results showed that the protease produced by Pseudomonas exhibited excellent heat resistance (Figure S2). After being treated at 140 °C for 5 s, 58.6% of the enzyme activity could still be retained. *Lactococcus* and *Chryseobacterium* also showed some enzyme residual activity, with the highest enzyme residual of 32.3% and 29.6%, respectively, after treatment at 140 °C for 5 s (Figure S2), which is similar to the fundings of Yuan et al. [17]. Furthermore, we observed that the lipase secreted by *Acinetobacter* demonstrated strong heat resistance, with the highest residual enzyme activity of 55.4% after treatment at 140 °C for 5 s. Previous studies have also confirmed the heat resistance of lipase produced by *Acinetobacter*, which enables it to survive in pasteurized milk [17]. The representative strains for the subsequent test were selected based on the strongest production of thermostable enzymes from four genera, including *P. fluorescens* (S3–4), *C. carnipullorum* (S8–14), *L. raffinolactis* (S8–29), and *A. guillouiae* (S7–15).

### 3.2. Physicochemical Properties of Milk Samples

#### 3.2.1. Protein and Fat Content

**Figure 1:**
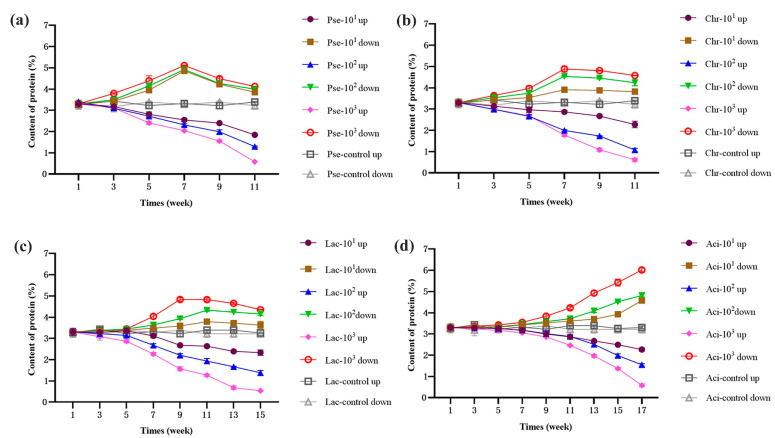
Changes of the upper and bottom protein content in UHT milk during storage. Note: (**a**–**d**) UHT milk added with *P. fluorescens*, *C. carnipullorum*, *L. raffinolactis*, and *A. guillouiae*, respectively.

Changes in protein and fat content can reflect the spoilage of UHT milk. To assess the hydrolysis effect of enzymes in milk, *P. fluorescens*, *C. carnipullorum*, *L. raffinolactis*, and *A. guillouiae* were added to UHT milk at different concentrations (10^1^, 10^2^, and 10^3^ cfu/mL) and stored at 25 °C. The protein content in the upper and bottom layers of milk during storage are illustrated in Figure 1. In general, the upper protein content decreased in milk contaminated by *P. fluorescens*, *C. carnipullorum,* and *L. raffinolactis*, while the bottom protein content initially increased and then decreased. However, in the milk with *A. guillouiae*, the upper protein content decreased and the bottom increased. The protein content in the upper and bottom of the control group showed no significant change under the storage condition at 25 °C, indicating that the hydrolysis of thermostable enzymes produced by psychrotrophic bacteria was the main factor affecting the protein content of UHT milk. Additionally, the changes in protein content in both the upper and bottom layers were more obvious with the concentration of strains added to the UHT milk. The changes in the upper and bottom proteins may be attributed to the actions of protease produced by psychrotrophic bacteria. Similar research was provided by Khusniati [24]. They found that the longer the storage times, the higher protease activities and protein degradation of milks contaminated by semi-purified *P. fluorescens*. Initially, the κ-casein is hydrolyzed by the protease, leading to a reduction In the stability of casein micelles and milk fat globules. Consequently, protein aggregation occurs, resulting in precipitation [25]. Subsequently, degradation of the bottom protein occurs, which could be associated with the presence of plasmin in milk [11]. Previous studies have indicated that plasmin and its plasminogen are primarily found in casein, while others are present in whey and fat globules [26,27]. The instability of plasmin is mainly influenced by the presence of thermostable enzymes produced by psychrotrophic bacteria, which negatively affect the stability of casein micelles [28]. Subsequently, plasmin released from casein enters the whey and selectively hydrolyzes αs-casein and β-casein, resulting in the formation of a casein by-product [11,29]. Consequently, there is an overall decrease in upper protein content in UHT milk. However, the changes observed in UHT milk contaminated by *A. guillouiae* were primarily attributed to the relatively weak protease production ability of *A. guillouiae*. This led to a lower transfer of plasmin, as confirmed by Zhang et al. [30].

**Figure 2:**
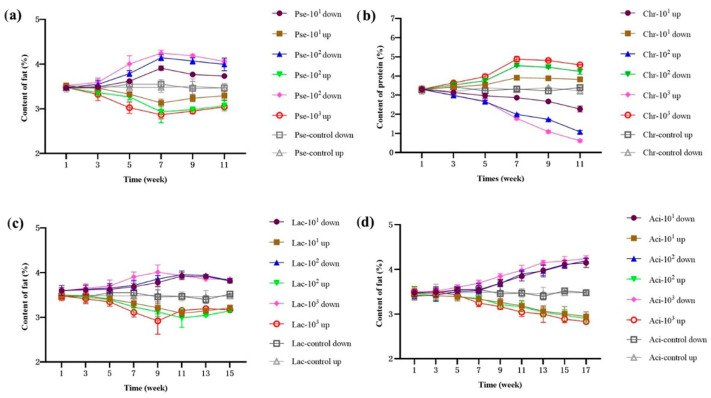
Changes of the upper and bottom fat content in UHT milk during storage. Note: (**a**–**d**) UHT milk added with *P. fluorescens*, *C. carnipullorum*, *L. raffinolactis*, and *A. guillouiae*, respectively.

Casein molecules are adsorbed onto the membrane of homogenized fat globules [31]. In the presence of protease, the structure of casein on the fat surface is damaged, causing the milk fat globules to gather and be wrapped in casein [32]. Plasmin in milk hydrolyzed αs-casein and β-casein into small pieces [30,33]. Consequently, the lipase produced by psychrotrophic bacteria can penetrate the fat globule membrane and act on triglycerides [34]. The concentration of fat in UHT milk, which was contaminated by *P. fluorescens*, *C. carnipullorum,* and *L. raffinolactis* during storage, demonstrated a pattern of initial decrease followed by an increase in the upper layer, while the lower layer exhibited an initial increase followed by a decrease, as illustrated in Figure 2. The main reason for the observed phenomenon was the action of the protease produced by psychrotrophic bacteria. Initially, this protease broke up the casein micelles, resulting in a decrease in the repulsive force between the milk fat globules. As a result, the fat globules aggregated but remained encapsulated in casein. Due to gravity, sedimentation occurred, leading to a decrease in the upper fat content and an increase in the bottom fat content. Subsequently, plasmin hydrolyzed αs-casein and β-casein. This hydrolysis caused the release of fat globules, which floated up due to their low density. Later, these fat globules were further hydrolyzed by lipase, similar to the findings of Zhang [35]. In the case of UHT milk contaminated by *A. guillouiae*, there was a decreasing trend in the upper fat content and an increasing trend in the bottom fat content. This phenomenon can be attributed to the limited lipase production capacity of *A. guillouiae*, which is consistent with the previous explanation.

#### 3.2.2. pH

**Figure 3:**
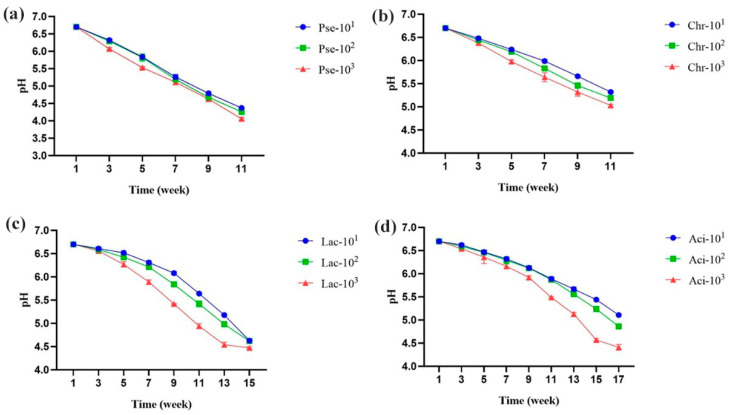
Changes in pH of UHT milk during storage. Note: (**a**–**d**) UHT milk added with *P. fluorescens*, *C. carnipullorum*, *L. raffinolactis*, and *A. guillouiae*, respectively.

The pH of all four milk samples decreased during storage, as shown in Figure 3. To the best of our knowledge, the four strains used have not been reported to have lactase production abilities. Thus, we speculated that the decrease in pH was primarily caused by lipid hydrolysis, which resulted in the production of free fatty acids and small organic acids. The rate of pH reduction during storage was found to be highest in *P. fluorescens*, followed by *C. carnipullorum*, *L. raffinolactis*, and *A. guillouiae*. Additionally, the hydrolysis rate of the four samples was found to be influenced by the concentration of the inoculated strains.

#### 3.2.3. Taste

**Figure 4:**
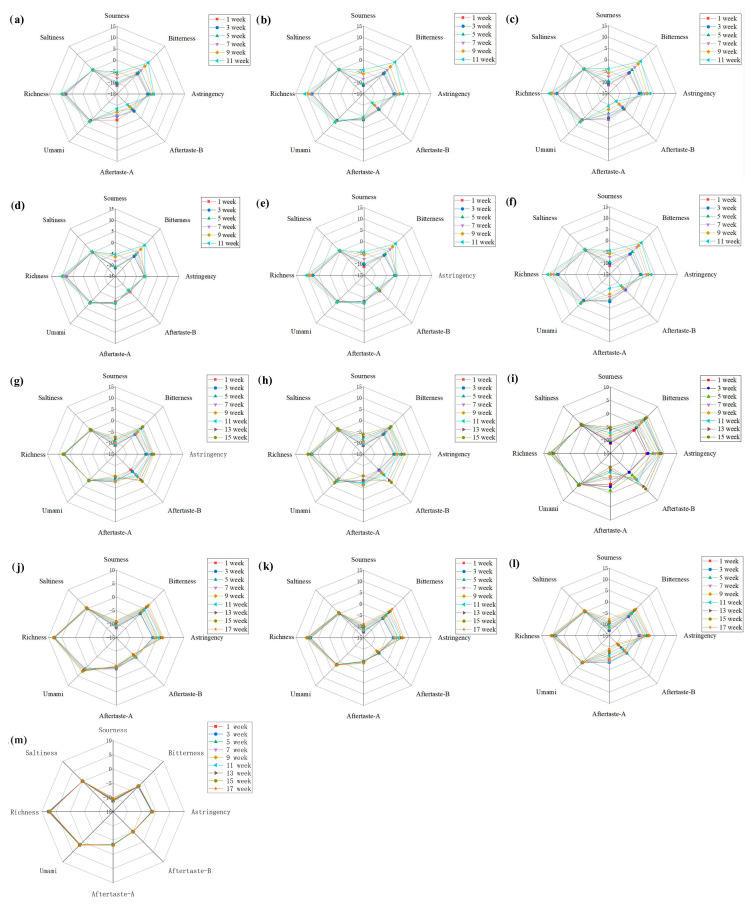
Radar map of milk taste changes during storage. Note: (**a**–**c**) the UHT milk added with 10^1^, 10^2^, 10^3^ cfu/mL *P. fluorescens*, (**d**–**f**) the UHT milk added with 10^1^, 10^2^, 10^3^ cfu/mL *C. carnipullorum*, (**g**–**i**) the UHT milk added with 10^1^, 10^2^, 10^3^ cfu/mL *L. raffinolactis*, (**j**–**l**) the UHT milk added with 10^1^, 10^2^, 10^3^ cfu/mL *A. guillouiae*, (**m**) the non-contaminated UHT milk.

Studies have shown that the quality of UHT milk can be affected by psychrotrophic bacteria during storage, leading to flavor deterioration [36]. A bitter taste in UHT milk is primarily caused by peptides of a specific molecular weight resulting from protease hydrolysis [37]. Additionally, a bitter taste can also be caused by low molecular aldehydes and ketones produced by lipase hydrolysis. The taste changes in UHT milk were analyzed using an electronic tongue (Figure 4), and it was found that bitterness was the main taste characteristic among the four groups of samples compared with non-contaminated samples. The bitter value showed a positive correlation with storage time and the concentration of the inoculated strains. Milk samples inoculated with *P. fluorescens* and *C. carnipullorum* showed more noticeable taste changes compared to the other two strains. However, there were no significant changes in saltiness and umami, and the variation pattern of the aftertaste value was complex. Richness, a taste after umami, showed an increasing trend during storage, which was mainly caused by free amino acids and peptides produced by proteolysis. Overall, the odor changes in the four samples were different, which could be attributed to variations in the degree of protein and fat hydrolysis. Wang et al. conducted a proteomics analysis and discovered that *Pseudomonas fluorescens* W3 negatively impacts the quality of UHT milk through the secretion of various proteases and peptidases [38]. The bitter taste of UHT milk has been attributed to the presence of certain bitter peptides, which are formed due to proteolytic reactions, thereby supporting our study.

#### 3.2.4. Volatile Compounds

The volatile compounds of UHT milk before and after spoilage are presented in Table 1. The addition of different species to milk resulted in changes in the number of volatile flavor, particularly alkanes. Milk samples contaminated with *P. fluorescens* and *C. carnipullorum* showed a significant increase in the number of alkanes, while the milk sample inoculated with *C. carnipullorum* showed a significant increase in the number of ketone compounds. Therefore, alkane and ketone compounds play a crucial role in the flavor of milk. The Maillard reaction, which usually accompanied heat treatment, has been reported to be associated with flavor compounds such as aldehydes and methyl ketones in UHT milk during storage [39]. Additionally, alkanes, alcohols, acids, and esters were produced by lipolysis [40].

### 3.3. Stability Evaluation

**Figure 5:**
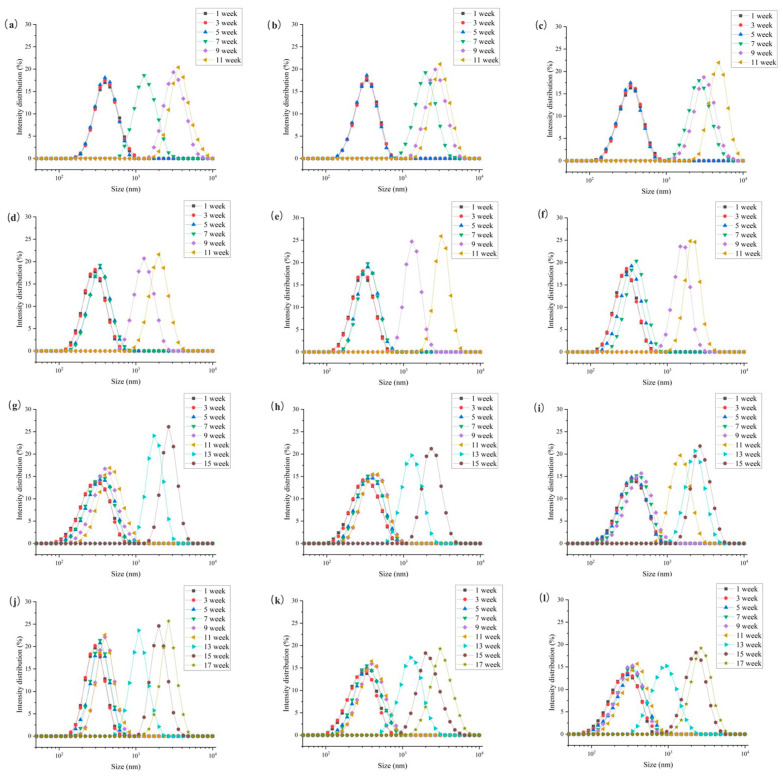
Particle size change in UHT milk during storage. Note: (**a**–**c**) the UHT milk added with 10^1^, 10^2^, 10^3^ cfu/mL *P. fluorescens*, (**d**–**f**) the UHT milk added with 10^1^, 10^2^, 10^3^ cfu/mL *C. carnipullorum*, (**g**–**i**) the UHT milk added with 10^1^, 10^2^, 10^3^ cfu/mL *L. raffinolactis*, (**j**–**l**) the UHT milk added with10^1^, 10^2^, 10^3^ cfu/mL *A. guillouiae*.

The composition of liquid milk comprises casein micelles and fat globules, with the protein particles being smaller in size compared to the fat globules [10]. As shown in Figure 5, the particle sizes of the four samples increased during the storage period, ranging from 10^2^ nm to 10^3^–10^4^ nm, which is supported by Boor [41]. The particle size distribution consistently exhibited a single peak, with the peak only slightly shifting to the right in the first 9 weeks, indicating an overlap between the peaks of casein and fat globules [30]. During this period, the casein micelles were smaller than the average size, while the fat globules were larger. Subsequently, the peak shifted significantly to the right, indicating that smaller and larger particles merged together to form larger particles [22,42]. In other words, the fat became trapped within the formed casein micelles [26]. As the concentration of the inoculum increased and the storage time was extended, the peak exhibited a rightward shift and the intensity of the peak became more pronounced. This phenomenon was also observed in a study by Francois et al. [4], who examined the impact of heat-resistant protease and plasmin produced by psychrotrophic bacteria on the stability of fat globulin in UHT milk.

**Figure 6:**
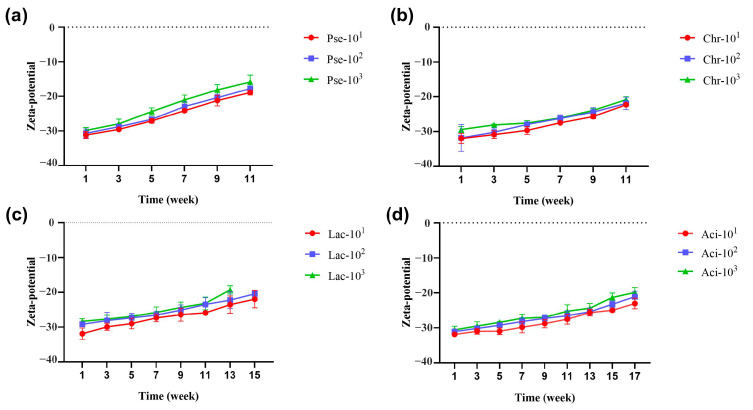
Zeta potential changes during milk storage. Note: (**a**–**d**) UHT milk added with *P. fluorescens*, *C. carnipullorum*, *L. raffinolactis*, and *A. guillouiae*, respectively.

The Zeta potential serves as an important indicator to characterize the stability of colloidal dispersion systems. In general, the Zeta potential is negative, and a higher absolute value indicates greater stability of the dispersion system. During storage at 25 °C, the absolute value of the potential for four samples exhibited a decreasing trend, as depicted in Figure 6. This suggests a decrease in the stability of the dispersion system, with all samples showing a correlation between stability and inoculated concentration. The sample contaminated by P. fluorescens exhibited the fastest change in stability, followed by *C. carnipullorum*, *L. raffinolactis*, and *A. guillouiae*. The variations among the four samples primarily stemmed from the corruption degree of the sample milk, which directly influenced the stability of the dispersion system. The stability of the dispersion system was influenced by the strains used, the concentration of inoculation, and the duration of storage.

**Figure 7:**
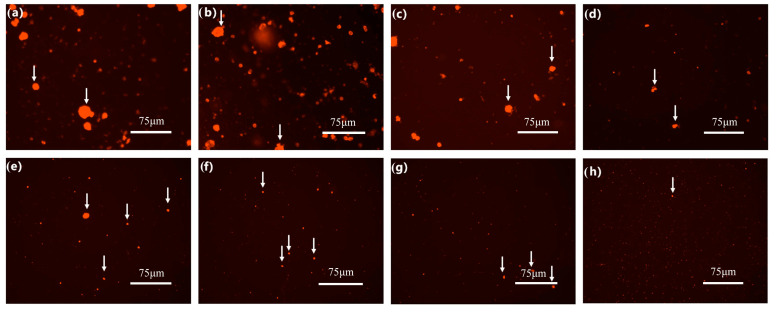
Microstructure of milk after hydrolysis. Note: In (**a**–**d**), the white arrows in images represent protein aggregates, and in (**e**–**h**), the white arrows in images represent milk fat globule aggregates; the ruler is 75 μm.

According to preliminary experiments, obvious whey separation occurred at 11, 11, 15, and 17 weeks, which indicated spoilage of the milk samples contaminated by *P. fluorescens*, *C. carnipullorum*, *L. raffinolactis*, and *A. guillouiae*, respectively. The protein aggregation in the samples contaminated by four strains showed varying degrees of change, as observed through an inverted fluorescence microscope (Figure 7). At this stage, although the milk samples were not completely spoiled, there were significant changes in their microstructure and the degree of spoilage varied among the four samples.

### 3.4. Metabolomic Analysis on the Changes of UHT Milk Quality

#### 3.4.1. Multivariate Statistical Discrimination of Milk Samples

**Figure 8:**
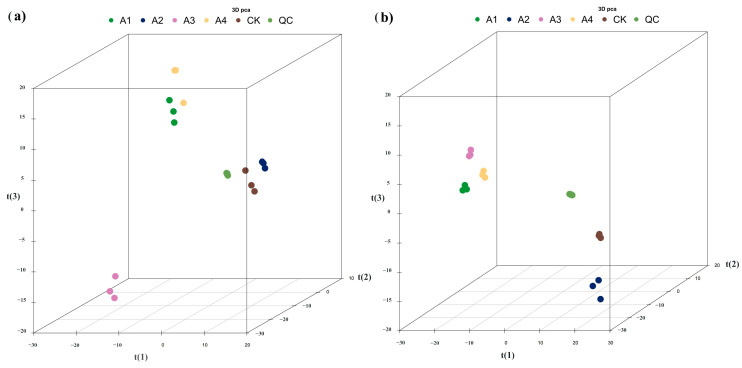
PCA score plot of samples under positive and negative ion separation. Note: (**a**) PCA score plot under positive ion separation, (**b**) PCA score plot under negative ion separation. t(1) represents principal component 1, t(2) represents principal component 2, t(3) represents principal component 3, and the aggregation degree of QC samples reflects the repeatability of the experiment.

An untargeted metabolomics-based approach was utilized to comprehensively investigate the metabolites that characterize different milk samples. The method employed UHPLC-ESI/QTOF mass spectrometry. Figure 8 displays the PCA analysis of the total samples under positive and negative ion conditions. Overall, PCA analysis was performed for each comparison group and the R2X of the PCA model, obtained through seven cycles of interactive verification, was close to one, indicating the stability and reliability of the model. The four sample groups obtained through HILIC (Hydrophilic Interaction Liquid Chromatography) chromatography separation conditions in the ESI+ and ESI− modes were relatively clustered within the group, with a good separation between groups. This suggests that samples within the same group had high similarity in metabolite composition, while different groups of samples exhibited significant differences in metabolite composition.

#### 3.4.2. Metabolites Identified in Milk Samples

**Figure 9:**
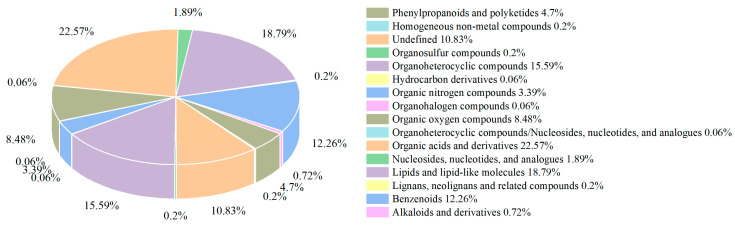
Percentage of identified metabolites in each chemical class.

**Figure 10:**
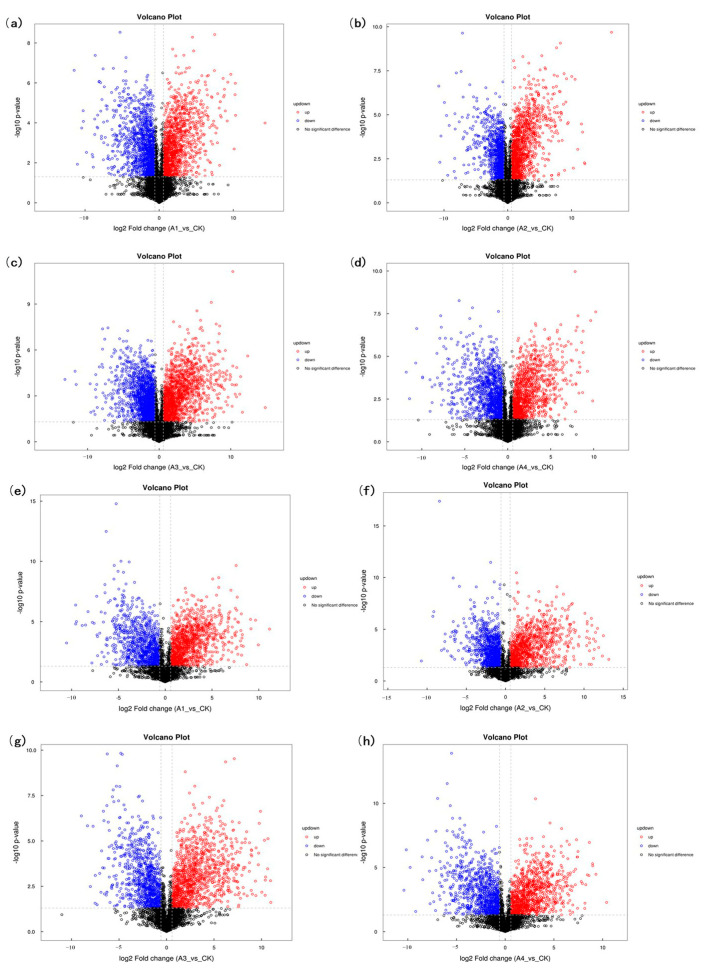
Differential protein volcano map. Note: (**a**–**d**) shows the volcano map of differentially expressed proteins between the A1–A4 group and the control group at positive ion mode. (**e**–**h**) shows the volcano plots of differentially expressed proteins between A1–A4 group and control group at negative ion mode. Red represents the up-regulated metabolite content, blue represents the down-regulated metabolite content, and black represents no difference in metabolite content.

A total of 1533 metabolites were identified using positive and negative ions. The classification, as shown in Figure 9, mainly consisted of organic acids and derivatives, lipids and lipid-like molecules, organic heterocyclic compounds, and benzene compounds. Among these, group A1, A2, A3, and A4 had 314, 317, 393, and 308 differential metabolites, respectively, indicating variations in the metabolic profiles of the different samples. The volcano diagram (Figure 10) visually displayed the differential metabolites between the four groups, revealing significant up-regulation and down-regulation compared to the control group. Compared with the blank group, a total of 781 metabolites were up-regulated and 752 metabolites were down-regulated in group A1 under positive and negative ions. A total of 767 metabolites were up-regulated and 766 metabolites were down-regulated in group A2 under positive and negative ions. A total of 851 metabolites were up-regulated and 682 metabolites were down-regulated in group A3 under positive and negative ions. A total of 775 metabolites were up-regulated and 758 metabolites were down-regulated in group A4 under positive and negative ions. Except the A3 group, the number of differential metabolites was similar among the other three groups, but their distribution varied.

Variable importance for the projection (VIP) is a metric that can indicate the degree of influence that metabolites have on various samples. Generally, a VIP value greater than one indicates significant contributions, and a higher value suggests a higher importance of the metabolite. Using a standard of VIP > 2 and *p* < 0.05, we identified 67, 64, 70, and 67 different metabolites that were significantly different between the A1, A2, A3, and A4 groups, respectively, compared to the control group (File S1). In the A1 group, a total of 23 metabolites exhibited up-regulation, while 44 metabolites displayed down-regulation. Similarly, within the A2 group, a total of 34 metabolites were up-regulated, while 30 were down-regulated. The A3 group exhibited 36 up-regulated metabolites and 34 down-regulated metabolites, whereas the A4 group had 27 up-regulated metabolites and 40 down-regulated metabolites. 

#### 3.4.3. KEGG-Enriched Pathways for Differential Metabolites

**Figure 11:**
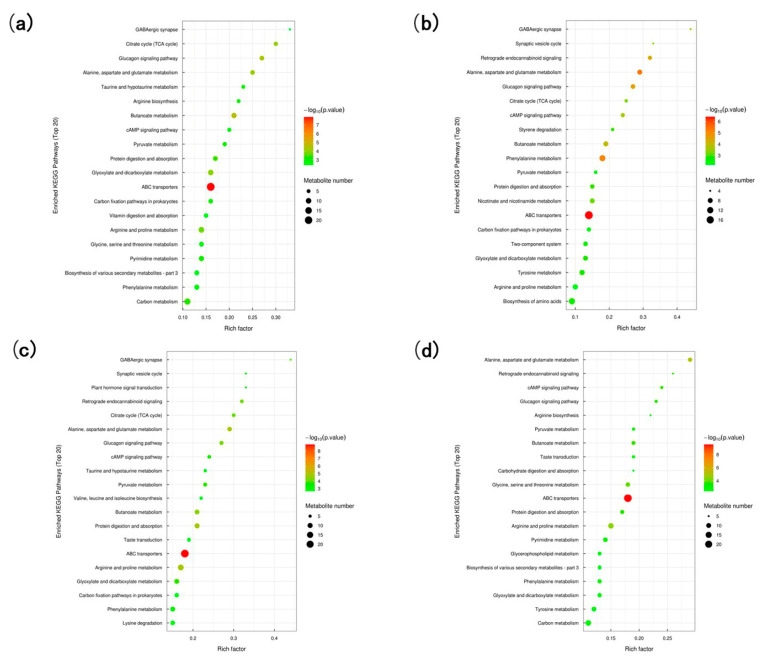
KEGG-enriched pathway map of A1, A2, A3, A4, respectively (**a**–**d**).

Metabolites within metabolome exhibit interconnectedness and do not exist independently. To gain a comprehensive understanding of the metabolic process of UHT milk contaminated by psychrotrophic bacteria, it is necessary to analyze the metabolic pathways. These pathways encompass energy metabolism, signal transmission, material transport, cell cycle regulation, and other relevant information during the storage of UHT milk contaminated by psychrotrophic bacteria. Figure 11 illustrates the enrichment diagram of the A1, A2, A3, and A4 pathways. Compared to the control group, the four groups exhibited the presence of ABC transporters, butanoate metabolism, and alanine, aspartate, and glutamate metabolism pathways. However, other metabolic pathways varied among the groups. Group A1, for example, showed the occurrence of glucagon signaling, citrate cycle, and glyoxylate and dicarboxylate metabolism pathways. Phenylalanine metabolism, and the glucagon signaling pathway and the retrograde endocannabinoid signaling pathway were predominantly observed in group A2. The metabolic pathways of arginine and proline, protein digestion and absorption, and the glucagon signaling pathway were mainly observed in group A3. Arginine and proline metabolism, glycine, serine, and threonine metabolism, and the cAMP signaling pathway were mainly observed in group A4. Although some metabolic pathways were shared among the four groups, the intensity of these pathways varied. In group A1, the ABC transporters were found to produce a total of 22 distinct metabolites. Additionally, the metabolism of butyrate resulted in the production of nine different metabolites, while the metabolism of alanine, aspartic acid, and glutamate yielded seven distinct metabolites. In group A2, ABC transporters produced nineteen different metabolites, butyrate metabolism produced eight different metabolites, and alanine, aspartic acid, and glutamic acid metabolism produced eight different metabolites. In group A3, ABC transporters produced twenty-four different metabolites, butyrate metabolism produced nine different metabolites, and alanine, aspartic acid, and glutamate metabolism produced eight different metabolites. Similarly, in group A4, ABC transporters produced twenty-four different metabolites, butyrate metabolism produced eight different metabolites, and alanine, aspartic acid, and glutamate metabolism produced eight different metabolites. The varying contents of metabolites in different groups exhibited distinct trends, either increasing or decreasing. These findings suggest a strong correlation between the changes in UHT milk quality during storage and these specific metabolic pathways.

The identified differential metabolites in the four sample groups were found to be associated with ABC transporters and butanoate metabolism. Organic acids and their derivatives, as well as lipids and lipid-like molecules, accounted for a significant proportion. Recent studies have shown that certain members of the ABC family are involved in lipid metabolism [43]. Butanoate is a natural nutrient found in foods like butter and milk [44] and is mainly present in the form of three acyl glycerides. The presence of butanoate metabolism in milk is due to the enzymatic breakdown of fats by lipases produced by psychrotrophic bacteria. Part of the released butyric acid contributes to the development of a rancid flavor, while the remaining portion undergoes subsequent reactions leading to the formation of various organic acids, derivatives, lipids, and lipid-like molecular metabolites. This finding aligns with previous volatile matter detection results. The heat-resistant protease produced by psychrotrophic bacteria in milk can simultaneously disrupt the casein micelle, which is located on the surface of fat globules. This disruption results in the liberation of milk fat globules into the emulsion system, where they undergo decomposition by lipase. As storage time increases, the lipid metabolites continually change, resulting in the development of a rancid flavor, peculiar smell, and degradation of nutrients. Additionally, it was observed that all four samples exhibited involvement in the metabolic pathways of alanine, aspartate, and glutamate. Proteins undergo hydrolysis by heat-resistant protease enzymes, resulting in the breakdown of amino acids. During this process, glutamate plays a crucial role in the Maillard reaction, where it reacts with glucose to produce a distinct bitter almond flavor. Bitter peptides, produced through enzymatic hydrolysis of proteins, and the bitter almond flavor resulting from the Maillard reaction, are the main factors responsible for the distinct bitter taste of UHT milk. This finding is consistent with previous results obtained through electronic tongue analysis. The metabolism of the milk system is complex throughout the storage period. The changes in the quality of UHT milk, caused by thermostable enzymes produced by psychrotrophic bacteria, are closely connected to the metabolic pathways mentioned above.

## 4. Conclusions

The production of thermostable enzymes by psychrotrophic bacteria poses a significant challenge to the dairy industry. This study aimed to investigate the impact of four representative strains, known for their strong production of thermostable enzymes, on the quality of UHT milk. Although the spoilage caused by *P. fluorescens*, *C. carnipullorum*, *L. raffinolactis*, and *A. guillouiae* differed, the predominant taste in spoiled milk samples was bitterness, accompanied by the presence of ketones and acids as the main odor. During storage, the content of upper protein and pH decreased. Additionally, the stability of the milk system decreased significantly, and protein and fat gathered in different degrees. Metabolomics analyses indicated that the three key metabolic pathways, including ABC transporters, butanoate metabolism, and alanine, aspartate, and glutamate metabolism, may influence the flavor and nutrient composition of UHT milk. This study provides a preliminary exploration of the mechanism by which the thermostable enzymes produced by psychrotrophic bacteria affect milk quality, offering a theoretical foundation for future efforts to mitigate the economic losses caused by these bacteria in the dairy industry. However, further research is needed to investigate the interaction between thermostable enzymes and milk, as well as the aging gelation mechanism of milk.

## Figures and Tables

**Table 1:** Types and percentage of volatile components in liquid milk before and after hydrolysis.

Compounds	Control Group		Adding *P. fluorescens*		Adding *C. carnipullorum*		Adding *L. raffinoltis*		Adding *A. guillouiae*	
	Kind	Relative Concentration (%)	Kind	Relative Concentration (%)	Kind	Relative Concentration (%)	Kind	Relative Concentration (%)	Kind	Relative Concentration (%)
Alkanes	6	14.41	15	17.43	15	16.00	8	15.30	9	6.35
Ketone	4	77.07	6	68.11	8	69.83	6	69.30	7	89.77
Aldehydes	-	-	1	0.25	1	0.14	-	-	-	-
Acid	-	-	3	0.48	2	0.22	2	0.08	3	0.15
Alcohol	-	-	2	3.52	4	4.10	-	-	2	0.42
Esters	-	-	-	-	1	0.13	-	-	-	-
Alkene	2	3.02	1	3.94	1	3.31	3	4.59	1	1.10
Benzene	5	9.22	4	6.28	4	6.13	5	10.73	4	2.15
Others	-	-	-	-	-	-	-	-	1	0.06

Note: “-” indicates not detected.

## Data Availability

The datasets used and/or analyzed during the current study are available from the corresponding author on reasonable request.

## Supplementary Materials

The following supporting information can be downloaded at: https://www.mdpi.com/article/10.3390/foods12203752/s1, File S1: significantly different metabolites between A1, A2, A3, A4 group and control group, respectively; Figure S1: The hydrolysis capacity of strains. Proteolytic diameters of *Pseudomonas* spp. (a), *Chryseobacterium* spp. (b), and *Lactococcus* spp. (c). Lipid-soluble diameters of *Pseudomonas* spp. (d), and *Acinetobacter* spp. (e); Figure S2: Thermotolerance of protease from *Pseudomonas* spp. (a), *Chryseobacterium* spp. (b), and *Lactococcus* spp. (c). Thermotolerance of lipase from *Pseudomonas* spp. (d), and *Acinetobacter* spp. (e); Table S1: main components and contents of aseptic whole milk.
