# Supplementary material for: Effect of Thermostable Enzymes Produced by Psychrotrophic Bacteria in Raw Milk on the Quality of Ultra-High Temperature Sterilized Milk
Source: Foods. 2023 Oct 12;12(20):3752. doi: 10.3390/foods12203752 (PMC10606520; doi:10.3390/foods12203752)
Supplement: Supplementary file 1 [file foods-12-03752-s001.zip › Supplementary figures and tables.pdf]

**Figure captions**

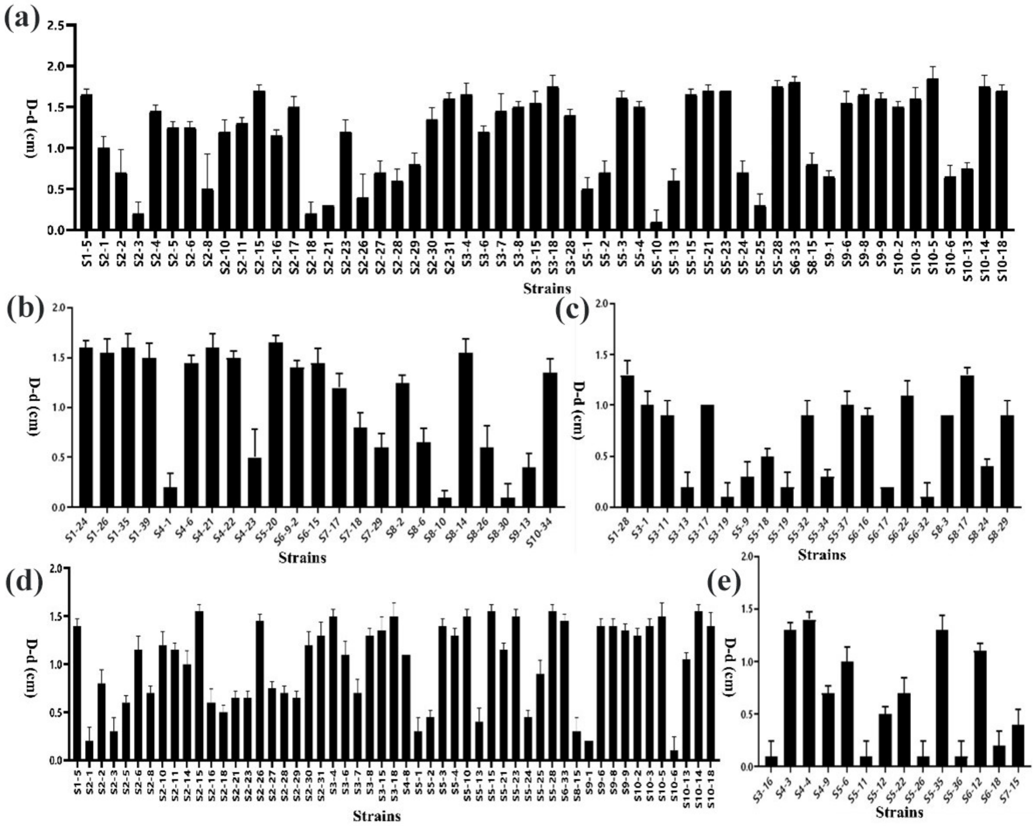


**Figure S1**

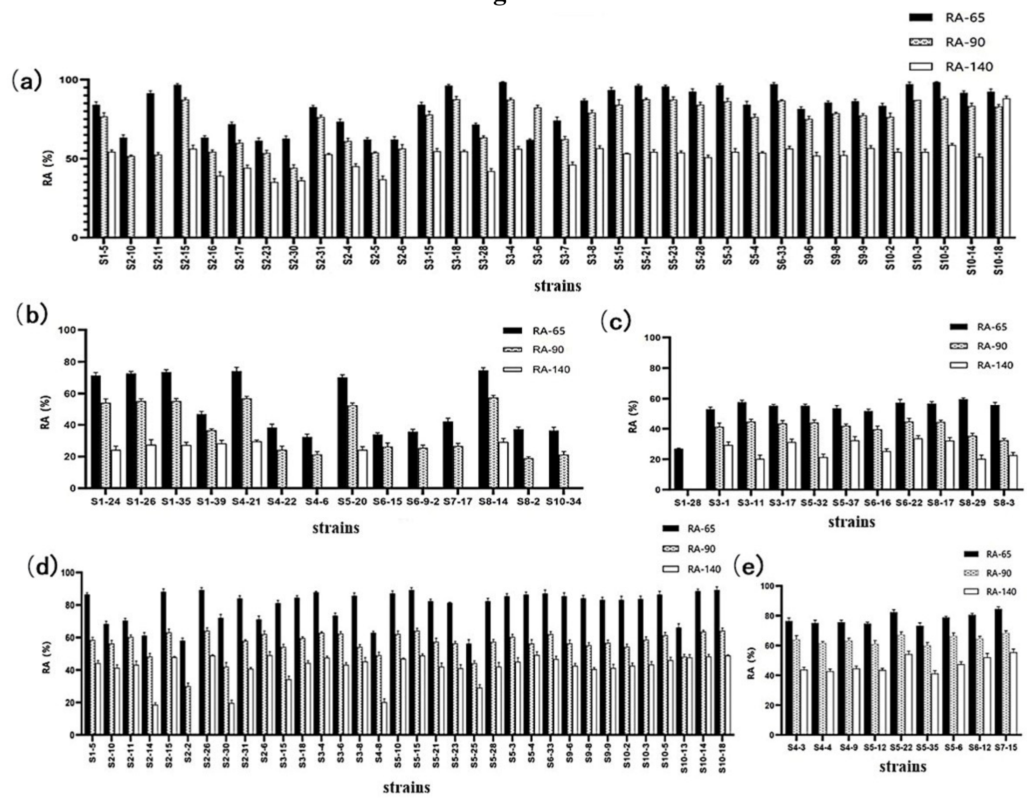


**Figure S2**

Table S1 The main components and contents of sterilized milk

| Genus | Species | Number | Serial number |
|---|---|---|---|
| *Pseudomonas* | *fluorescens* | 15 | S2-15 S2-26 S3-4 S3-18 S5-3 S5-10 S5-15 S5-21 S5-23 S5-28 S6-33 S10-3 S10-5 S10-14 S10-18 |
| | *azotoformans* | 9 | S1-5 S2-31 S3-8 S3-15 S5-4 S9-6 S9-8 S9-9 S10-2 |
| | *lurida* | 8 | S2-2 S2-3 S2-8 S2-18 S2-21 S2-27 S2-28 S2-29 |
| | *psychrophila* | 6 | S5-1 S5-2 S5-13 S5-24 S8-15 S9-1 |
| | *fragi* | 4 | S2-1 S2-5 S2-16 S2-23 |
| | *cedrina* | 4 | S2-4 S2-17 S3-7 S3-28 |
| | *lundensis* | 4 | S2-6 S2-10 S2-11 S3-6 |
| | *mandelii* | 4 | S2-12 S4-16 S4-28 S4-33 |
| | *fulva* | 3 | S4-11 S4-13 S4-31 |
| | *anguilliseptica* | 2 | S1-25 S1-36 |
| | *extremorientalis* | 2 | S2-13 S2-22 |
| | *putida* | 2 | S2-14 S4-8 |
| | *jessenii* | 1 | S2-25 |
| | *gessardii* | 1 | S2-30 |
| | *veronii* | 1 | S5-8 |
| | *maritimus* | 1 | S10-6 |
| | *brenneri* | 1 | S10-13 |
| *Chryseobacterium* | *carnipullorum* | 6 | S1-24 S1-26 S1-35 S4-21 S5-20 S8-14 |
| | *Chryseobacterium sp.* | 6 | S8-10 S8-16 S8-27 S8-30 S8-31 S9-12 |
| | *scophthalmum* | 4 | S4-6 S4-22 S6-15 S6-9-2 |
| | *indologenes* | 2 | S7-17 S7-29 |
| | *balustinum* | 2 | S8-6 S8-26 |
| | *chaponense* | 1 | S1-39 |
| | *aahli* | 1 | S4-1 |
| | *formosense* | 1 | S4-23 |
| | *xixisoli* | 1 | S7-18 |
| | *indoltheticum* | 1 | S8-2 |
| | *joostei* | 1 | S9-13 |
| | *haifense* | 1 | S10-36 |
| | *anthropi* | 1 | S10-34 |
| *Lactococcus* | *raffinolactis* | 17 | S3-1 S3-11 S3-13 S3-17 S5-9 S5-18 S5-19 S5-32 S5-34 S5-37 S6-16 S6-17 S6-22 S8-3 S8-17 S8-24 S8-29 |
| | *Lactococcus sp.* | 2 | S1-2 S1-19 |
| | *piscium* | 1 | S1-28 |

| | | | |
|---|---|---|---|
| | *lactis* | 1 | S3-19 |
| | *laudensis* | 1 | S6-32 |
| *Acinetobacter* | *guillouiae* | 7 | S4-3 S4-4 S4-9 S5-6 S5-11 S5-12 S5-35 |
| | *johnsonii* | 7 | S3-16 S5-22 S5-26 S5-36 S6-12 S6-18 S7-15 |
| | *albensis* | 4 | S2-20 S2-25 S8-1 S8-7 |
